# Differential Pulse Voltammetric Analysis of Cannabidiol on Fluorine-Doped Tin Oxide (FTO) Electrode Interface: Quantification in Pharmaceutical Oil with Assessment of Electrode Surface Stability

**DOI:** 10.3390/s26154893

**Published:** 2026-08-03

**Authors:** Mario Romero, Eliana Rocha Fontalvo, María L. Ospina-Castro, Victoria A. Arana, Carlos Mario Meléndez, Jolián Andrés Vargas Álzate, Andrea Ramos-Hernández

**Affiliations:** 1 Grupo de Investigación Química Supramolecular Aplicada, Programa de Química, Facultad de Ciencias Básicas, Universidad del Atlántico, Carrera 30 No. 8-49, Puerto Colombia 081007, Colombia; marioromero@mail.uniatlantico.edu.co (M.R.); emariarocha@mail.uniatlantico.edu.co (E.R.F.); mariaospina@mail.uniatlantico.edu.co (M.L.O.-C.); 2 Grupo de Investigación Ciencias, Educación y Tecnología—CETIC, Programa de Química, Facultad de Ciencias Básicas, Universidad del Atlántico, Carrera 30 No. 8-49, Puerto Colombia 081007, Colombia; victoriaarana@mail.uniatlantico.edu.co; 3 Grupo de Investigación en Química Orgánica y Biomedicina, Programa de Química, Facultad de Ciencias Básicas, Laboratorio de Investigación en Biología Molecular, Universidad del Atlántico, Carrera 30 No. 8-49, Puerto Colombia 081007, Colombia; carlosmelendez@mail.uniatlantico.edu.co; 4 CUBIKAN GROUP SAS, Finca Veracruz, Vereda La Diamantina—km 10 vía La Argelia, Ansermanuevo 762018, Colombia; info@cubikangroup.com

**Keywords:** cannabidiol, electroanalysis, FTO, electrochemical sensor, significance tests

## Abstract

This work presents the use of an unmodified fluorine-doped tin oxide (FTO) electrode as an electrochemical sensor for the stable and highly reproducible detection of cannabidiol (CBD) in an oral pharmaceutical formulation (CBD-OS). This was achieved using an electrochemical methodology with 0.1 M LiClO_4_ in acetonitrile as the electrolyte medium. The oxidation process of CBD on the FTO was extensively studied using cyclic voltammetry to analyze the influence of forced convection during the experiment. Quantification was performed by differential pulse voltammetry with standard addition to the dissolved CBD oral oil (CBD-OS), which allowed for the determination of a detection limit of 4.8 µM and a correlation coefficient R^2^ = 0.9989. Statistical tests indicated no significant differences between the proposed electrochemical methodology and HPLC. The FTO electrode exhibited high reproducibility, withstanding more than 20 consecutive additions to the same electrode without loss of response, thanks to its intrinsic stability and the strategic use of forced convection. The use of an unmodified electrode enabled the development of a simple, reproducible, robust, and cost-effective analytical methodology for CBD analysis in pharmaceutical samples.

## 1. Introduction

In recent years, cannabis has gained worldwide recognition for its therapeutic, medicinal, and recreational potential. This plant, belonging to the genus Cannabis, contains a wide variety of compounds of interest, most notably terpenes and phytocannabinoids. Among the latter, cannabidiol (CBD) is one of the most relevant non-psychoactive phytocannabinoids of *Cannabis sativa* L., extensively studied for its therapeutic properties in the treatment of neurological disorders, anxiety, inflammation, and chronic pain [1,2,3,4].

Determining CBD in commercial samples is crucial today due to the rapid growth of the cannabis-derived products market and the wide variety of available formulations, including oils, extracts, cosmetics, food, and therapeutic products. The actual CBD concentration directly influences the product’s efficacy, safety, and economic value, making its reliable quantification essential for both regulatory compliance and ensuring transparency for consumers. Therefore, having robust, rapid, and reproducible analytical methods enables the verification of authenticity, the detection of adulteration, and the evaluation of overall quality in commercial products, thus providing indispensable scientific support for industry and regulatory agencies [5,6,7,8,9].

Currently, the identification and quantification of cannabinoids are performed using high-performance analytical techniques such as high-performance liquid chromatography (HPLC), gas chromatography (GC), mass spectrometry (MS), and their combinations (GC-MS, LC-MS), as well as NMR spectroscopy. Although these techniques are highly accurate and reliable, they have significant disadvantages, including high cost, the use of large quantities of solvents, the need for specialized personnel, and lengthy analysis times [9,10,11].

In this context, electroanalytical techniques represent efficient, cost-effective, and environmentally friendly alternatives for determining CBD levels. These techniques take advantage of the electroactivity of the phenolic group present in the CBD structure [12,13]. Additionally, electrochemical instrumentation is quite versatile. Efforts are currently underway to miniaturize sensors and biosensors, aiming to develop portable devices that provide reliable analytical results [14]. Recent research in this field focuses on modified electrode surfaces that enable electroanalytical techniques to quantify cannabinoids in different matrices. However, in some cases, these modifications are specific, such as the use of graphene quantum dots (GQDs) [15] and molecular-imprinted mesoporous Pt-Ir surfaces [16], potentially impacting the reproducibility of the methods used to create these surfaces. In other cases, analyte separation processes are required before performing electrochemical measurements [17]. Therefore, it remains a challenge to develop electroanalytical methods that allow for the quantification of cannabinoids through simple processes, without complex sample preprocessing, using electrodes that are easy to obtain and reproduce on a larger scale, and with good analytical quality parameters, as other authors indicate [18].

In this sense, the FTO electrode, normally used for the study of materials focused on electronic applications, such as photovoltaic cells, among others [19,20,21], has recently aroused increasing interest in electroanalytical applications [22] due to its favorable physicochemical properties, including high electrical conductivity, transparency, chemical stability, and resistance to surface passivation processes [23,24]. These characteristics enable reproducible electrochemical responses across a wide anodic potential window, making it particularly suitable for studying the oxidation processes of compounds with a resorcinol core such as CBD. This contrasts with metallic or carbon electrodes; the FTO has a homogeneous surface and is easily renewable through simple cleaning procedures [25,26,27].

Taking the above into account, in this work, an electroanalytical method for the detection and quantification of CBD was developed using unmodified FTO electrodes and potentiodynamic electrochemical techniques, including cyclic (CV) and differential pulse voltammetry (DPV). This approach leverages the electrochemical stability, conductivity, and compatibility of FTO with organic media to achieve a sensitive and reproducible analytical response. The proposed method was specifically designed for the electroactive characterization and quantitative analysis of compounded medicinal cannabis formulations.

## 2. Materials and Methods

### 2.1. Reagents

Standard CBD crystals (98% purity, Cayman Chemical, Ann Arbor, MI, USA) were used to prepare standard solutions. Solvents included methanol (ACS reagent, ≥99.8%), acetonitrile (ACN) (for HPLC LiChrosolv^®^), Tetrahydrofuran (THF) (for HPLC LiChrosolv^®^ ≥ 99.9%) from Merck, KGaA, Darmstadt, Germany and absolute ethyl alcohol (ACS grade, ≥99.9%, J.T. Baker, Phillipsburg, NJ, USA). LiClO_4_ (ACS reagent, ≥95%) and tetrabutylammonium hexafluorophosphate (TBAPF_6_) (≥99.99%), both from Merck, KGaA, Darmstadt, Germany, were used as electrolytes. Additionally, a commercially compounded formulation of CBD isolate (318 mM or 100 mg/mL) in extra-virgin olive oil was analyzed.

### 2.2. Prepared Solutions

The standard CBD solution (3.2 mM or 1.0 mg/mL) was prepared from commercial crystals in methanol; the electrolytic solutions consisted of 0.1 M LiClO_4_ in ethanol and ACN, and 0.1 M TBAPF_6_ in ACN to optimize the electrochemical conditions for the characterization and quantification of CBD. Additionally, a solution containing 150 µM CBD in 0.1 M LiClO_4_ in ACN was prepared for redox-process characterization.

### 2.3. Electrochemical Characterization of CBD

**Figure 1:**
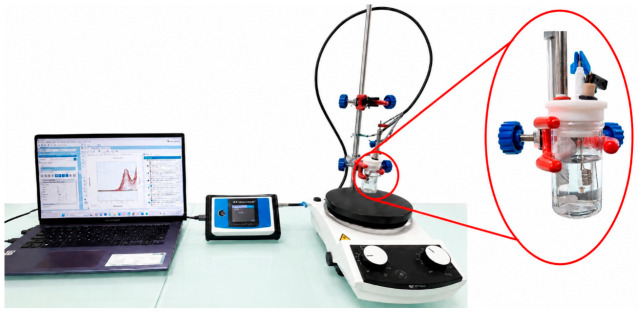
Working setup used for the construction of the calibration curve and electroanalytical measurements using an electrochemical cell with three electrodes.

Solutions of 0.3 mM (0.1 mg/mL) CBD were prepared in the electrolytic systems detailed above; they were analyzed using potentiodynamic methods, and various parameters were systematically evaluated to identify the optimal conditions for CBD oxidation. These factors included electrode surface (Pt, Au, Graphite, and FTO), potential windows, and scan rate. The CBD redox process was carried out by performing 25 scans per CV and per PDV. All measurements were performed using a PalmSens 4 potentiostat/galvanostat (PalmSens BV, Houten, The Netherlands) controlled by PSTrace 5.9 software and a three-electrode system consisting of: an Ag/AgCl reference electrode, a spiral platinum auxiliary electrode, and, as a working electrode, unmodified FTO with a geometrically bounded area of 0.5 cm^2^ (See Figure 1).

Before its use in electrochemical measurements, the FTO substrate underwent a cleaning protocol using sonication in a beaker. The procedure consisted of three sequential stages: initially, sonication in ultrapure water; subsequently, in acetone; and finally, in n-propanol. In each stage, the substrate was subjected to an ultrasonic bath at 37 kHz and a power of 540 W for 15 min. This process aims to eliminate surface impurities that could interfere with electrochemical measurements. This cleaning protocol for the FTO substrate has been reported for other applications [19,28].

### 2.4. Calibration Curve Construction

For the construction of the calibration curve, 10.00 mL of the electrolytic solution (LiClO_4_ 0.1 M in ACN) were transferred to a 20 mL capacity electrochemical cell. Additions of 100 μL of the CBD standard (3.2 mM, in methanol) were made, followed by magnetic stirring for 5 min at 260 rpm (MS-H280-Pro stirrer, DLAB, Beijing, China). After this time, the solution was allowed to stand for 30 s. The electrochemical measurement was performed using the following experimental parameters: initial potential (*E_i_*) of 0.5 V, final potential (*E_ƒ_*) of 2.0 V, potential step (Δ*E*) of 0.01 V, pulse amplitude (Δ*E_p_*) of 0.006 V, pulse duration (*t_p_*) of 0.1 s, and scan rate of 0.05 V/s. This experimental process was carried out interspersed with 20 additions of the CBD standard solution. All measurements were performed in triplicate to evaluate the repeatability of the method. The calibration curve was obtained by graphical analysis of the relationship between peak current (*I_p_*) and CBD concentration at each addition.

Once the series of additions necessary for constructing the calibration curve (20 electrochemical measurements) was completed, the FTO was subjected again to the cleaning protocol described above to remove adsorbed residues from its surface and restore its electrochemical activity. Subsequently, the clean electrode was reused to repeat the complete sequence of additions and measurements, allowing the replicates reported in this study to be obtained (*n* = 3).

### 2.5. Analysis of a Commercial Pharmaceutical Sample CBD-OS

**Figure 2:**
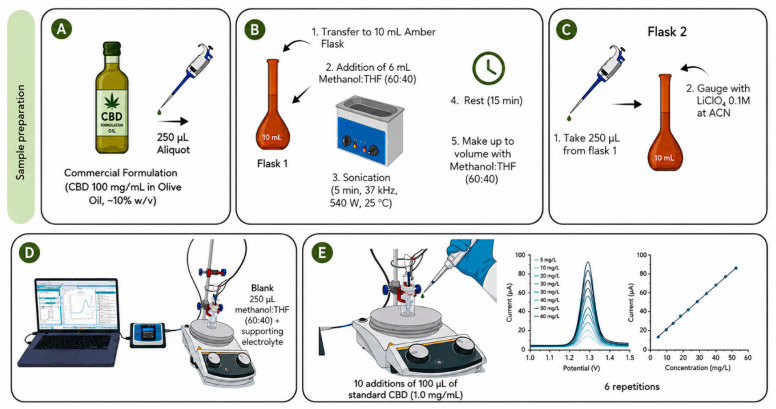
Experimental workflow for the electrochemical determination of cannabidiol (CBD) in pharmaceutical oil samples (image generated with AI support). The arrows indicate the sequence and direction of the experimental procedure. (**A**) Sample collection. (**B**) Primary dilution. (**C**) Secondary dilution. (**D**) Differential pulse voltammetry (DPV) measurements. (**E**) Quantification by the standard addition method.

For sample preparation, 250 µL of the initial formulation (nominal concentration of 318 mM or 100 mg/mL of CBD in olive oil, equivalent to 10% *w*/*v*) was transferred to a 10.00 mL amber volumetric flask. Overall, 6 mL of methanol:THF (60:40) was added; this mixture was used to dissolve the lipid matrix completely. The solution was sonicated for 5 min at 37 kHz, 540 W, and a controlled temperature of 25 °C (Elmac^®^ Elmasonic P 60 H^®^, Singen, Germany). The solution was then allowed to stand for 15 min at room temperature for stabilization and brought to the final volume with the methanol: THF (60:40) mixture, resulting in a primary dilution of 1:40. From this solution, 250 µL were taken and further diluted to 10.00 mL in another amber volumetric flask with the working electrolyte solution, 0.1 M LiClO_4_ in ACN (secondary dilution of 1:40), resulting in a total dilution factor of 1600. The final solution was quantitatively transferred to the electrochemical cell for differential pulse voltammetry measurements. The blank solution was prepared by adding 250 µL of methanol:THF (60:40) to a 10.00 mL volumetric flask and bringing the final volume to 10.00 mL with the supporting electrolyte described above (see Figure 2).

For the quantitative analysis of the commercial sample, 10 additions of 100 μL of the 3.2 mM standard CBD solution were made, following the same experimental protocol described in the Section 2.4, with a total of 6 repetitions.

### 2.6. Determination of the Limit of Detection and Quantification

To evaluate the limits of detection (LoD) and quantification (LoQ), 10.00 mL of the electrolytic solution (0.1 M LiClO_4_ in ACN) was prepared as an analytical blank. Ten consecutive measurements of this solution were performed using differential pulse voltammetry under the same instrumental conditions used to construct the calibration curve, to characterize the baseline and quantify the electroanalytical noise of the system. From these records, the standard deviation of the blank responses (*σ*) was calculated, which was subsequently used in the equations LoD = (3.3 × *σ*)/m and LoQ = (10 × *σ*)/m, where m is the slope of the calibration line obtained in the previous step [29,30].

### 2.7. Chromatographic Analysis

Chromatographic analysis was performed using an LC-4000 high-performance liquid chromatograph (HPLC) equipped with ChromNAV software (version 2.0), coupled with a UV-4075 ultraviolet-visible detector (Jasco, Tokyo, Japan) capable of capturing two wavelengths, and an Ascentis Express column (C18, 3.0 × 150 mm, 2.7 µm); Supelco, Bellefonte, PA, USA. A gradient elution system was used with water and acetonitrile (both HPLC grade) in a 40:60 (*v*/*v*) ratio as mobile phases A and B, respectively, both acidified with 0.08% formic acid. The gradient program was conducted as follows: 0–1 min 60% mobile phase B, 1–6.5 min 95% mobile phase B, 6.5–7.5 min 100% mobile phase B, 7.5–8.0 min 100% mobile phase B, 8.0–9.0 min 60% mobile phase B, and 9.0–12.0 min 60% mobile phase B, at a flow rate of 1.0 mL/min and column temperature of 45 °C, with detection at 214 nm. Sample preparation for analysis was performed by weighing 30 mg of the commercial formulation, adding 3 mL of methanol: THF (60:40, *v*/*v*) to a 5.00 mL volumetric flask, sonicating for 5 min, allowing to stand for 15 min, and then diluting to volume with the same diluent. From this solution, 1.0 mL aliquots were taken, added to 10.00 mL volumetric flasks, diluted to volume with a methanol: THF mixture (60:40), and then shaken.

### 2.8. Statistical Analysis of Electrochemical vs. HPLC Methods for Evaluating CBD Content in Commercial Pharmaceutical Formulations (CBD-OS)

To validate the results obtained using DPV, a comparative analysis was performed with HPLC, the reference methodology for traditional CBD quantification. The same sample, evaluated electrochemically (a 100 mg/mL CBD formulation in an olive oil matrix), was analyzed according to the sample preparation protocol. Six replicates were performed for each methodology (*n*_1_ = 6 = *n*_2_, equivalent to 10 degrees of freedom in this statistical analysis). The comparison between the two methodologies was carried out by statistical analysis of variance using the Fisher test (F), to evaluate the homogeneity of the experimental variances (*s*^2^) (F_calculated_ < F_critical_, *α* = 0.05, with degrees of freedom (df) df_electrochemical_ = df_HPLC_ = 5). Subsequently, the Student’s *t*-test for two experimental means with homogeneous variances was applied to determine if there are significant differences between the means obtained by both techniques (*t*_calculated_ < *t*_critical_, *α* = 0.05, at 10 df) [31].

## 3. Results and Discussion

The electrochemical behavior of CBD was studied using CV and DPV on an unmodified FTO electrode. Cyclic voltammetry (CV) was used in the exploratory stage to identify the oxidation potential of CBD and study the nature of the redox process. As a complementary assessment, the electrochemical behavior of the system was also investigated using differential pulse voltammetry (DPV). In the preliminary stage, the nature of the working electrode (Pt, Au, Graphite, and FTO) and the composition of the electrolyte solution were optimized by evaluating different supporting electrolytes and solvents (see Supplementary Material Figures S1 and S2). FTO showed a better electrochemical response in a solution of 0.1 M LiClO_4_ in ACN. This electrode was chosen as optimal despite requiring a higher potential than the other surfaces studied, which, although requiring lower potentials, also showed high ease of modification, in agreement with other authors [32], compromising the interface for CBD quantification and potentially affecting the analytical quality of the measurements. In this scenario, FTO showed better voltammetric resolution and the highest electrochemical stability, which is crucial for establishing a reproducible electroanalytical method. For this reason, these optimal conditions were used in all the experiments presented in this work.

**Figure 3:**
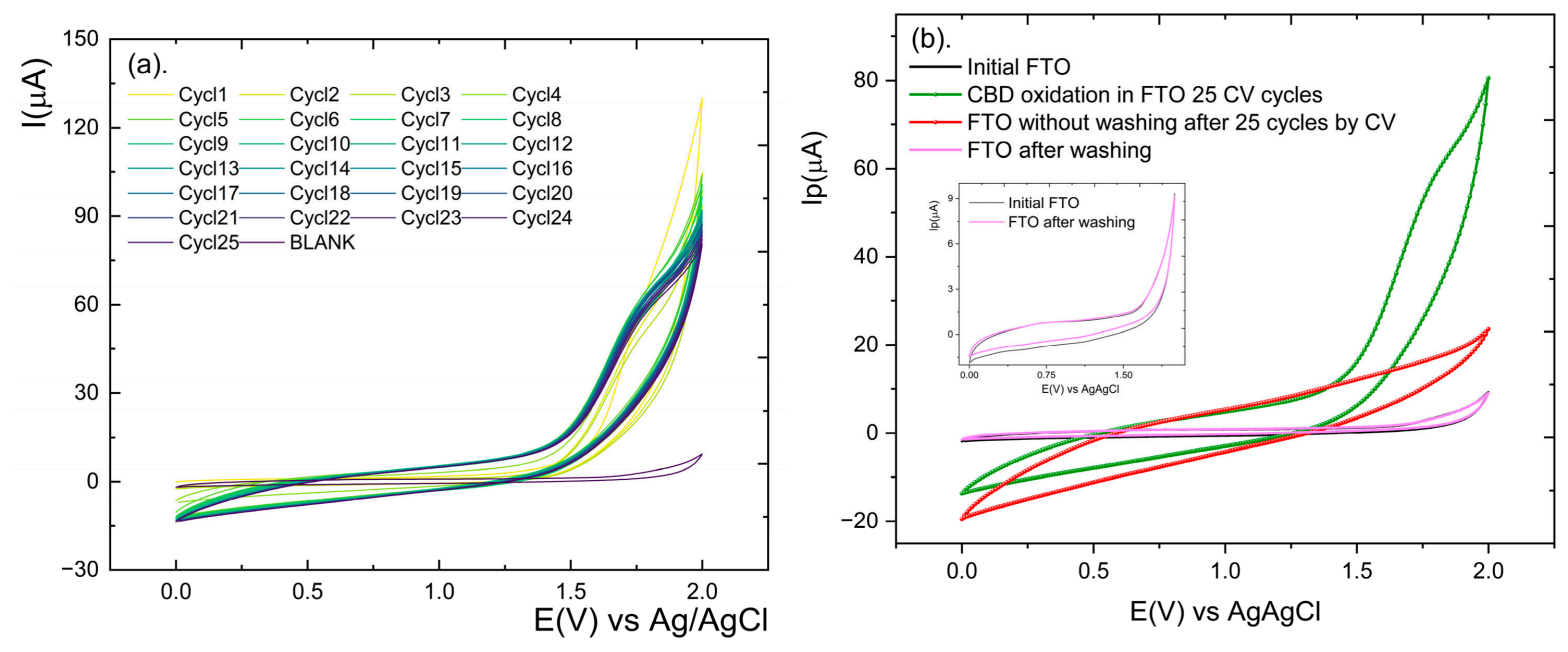
(**a**) Electrochemical oxidation of 150 µM CBD at the CBD/FTO interface by cyclic voltammetry (25 consecutive cycles) in 0.1 M LiClO_4_/ACN. (**b**) Cyclic voltammetric responses of the FTO electrode before CBD oxidation, after CBD oxidation, the 25th cyclic voltammogram recorded during the electrochemical oxidation of CBD, and after ultrasonic cleaning. The inset shows an enlarged comparison of the voltammetric responses before CBD oxidation and after ultrasonic cleaning.

Figure 3a shows the cyclic voltammograms recorded for a CBD (150 µM) solution in 0.1 M LiClO_4_ in ACN, obtained by 25 consecutive cycles at a sweep rate of 0.1 V/s. During the first cycle, the oxidation process begins at approximately 1.35 V and reaches a maximum current around 1.65 V. In subsequent cycles, a progressive decrease in the anodic current is observed, ceasing to show appreciable variations from cycle 15 onward and maintaining a similar voltammetric profile. This behavior suggests that, during the first few cycles, a gradual modification of the FTO’s electroactive surface occurs, associated with the accumulation of species generated by CBD oxidation.

Considering that CBD possesses a resorcinol core susceptible to forming radical species during its electrochemical oxidation, it is reasonable to suggest that these intermediates may undergo coupling reactions leading to the formation of dimers, oligomers, and, under certain conditions, larger molecular structures. This interpretation is consistent with the findings of Bonechi et al. [33,34] and other authors [35], who demonstrated that this electrochemical oxidation is favored by radical coupling processes and the progressive formation of oligomeric products at the electrode–solution interface. Similarly, Belhadj Tahar and Savall observed that the electropolymerization of phenolic compounds on a vitreous carbon electrode reduces the electrochemical response, implying partial passivation of the electrode, without necessarily resulting in complete blocking of the electroactive surface [36].

In this case, although the decrease in current during the first few cycles is consistent with the formation of coupling species on the electrode surface, the stabilization of the voltammetric response observed from cycle 15 onward indicates that this surface modification does not continue to increase progressively under the experimental conditions used. Consequently, the results suggest that CBD oxidation leads to limited surface modification, rather than a continuously increasing passivation process. This behavior also agrees with what has been described for the electrochemical oxidation of substituted anisoles, where the formation of soluble oligomers or weakly adsorbed species allows for the preservation of a significant fraction of the electroactive surface, preventing complete electrode blockage and favoring the stabilization of the electrochemical response in successive cycles [37].

To assess whether the progressive decrease in observed current during the voltammetric cycles was associated with a change in the FTO surface, the electrochemical response was compared before and after CBD characterization (Figure 3b). To do this, once the 25 cyclic voltammetry cycles were completed (green curve), the electrode was rinsed with acetonitrile to remove weakly bound residues from the surface. It was subsequently characterized again in a 0.1 M LiClO_4_ solution in ACN (red curve). Compared to the initial response of the FTO (black curve), a clear change in the voltammetric profile is observed, confirming that the oxidation of CBD causes changes at the electrode–solution interface. However, after applying the sonication-assisted cleaning procedure described in the experimental section, the electrochemical response of the FTO was restored to nearly its initial state, as shown in the inset of Figure 3b. This result indicates that the surface modification induced during CBD oxidation does not correspond to an irreversible blocking of the electroactive surface, but rather to a reversible process associated with species generated during oxidation, which can be removed by a simple physical cleaning procedure.

**Figure 4:**
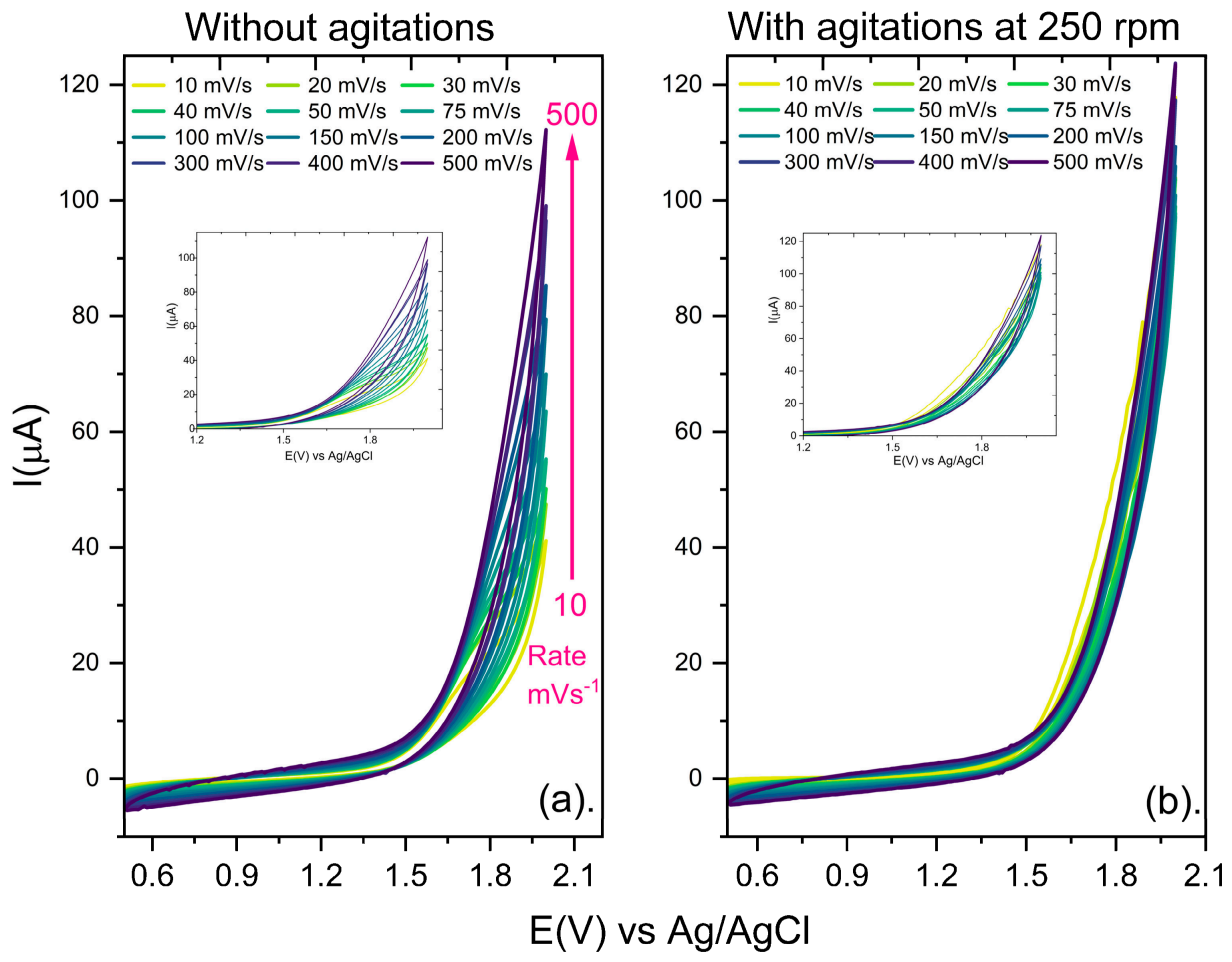
(**a**) Electrochemical oxidation of 150 µM CBD at different scan rates under stationary conditions in 0.1 M LiClO_4_/ACN using an FTO electrode. (**b**) Electrochemical oxidation of 150 µM CBD at different scan rates under hydrodynamic conditions in 0.1 M LiClO_4_/ACN using an FTO electrode.

Once the reversible nature of the surface modification was established, the influence of the scan rate on the CBD oxidation process was investigated using CV. Experiments were performed in a CBD solution (150 µM) in 0.1 M LiClO_4_/ACN, within a potential window of 0.5 to 2.0 V. Figure 4a shows the voltammograms obtained at scan rates between 10 and 500 mV/s under steady-state conditions. In contrast, Figure 4b shows the same study under constant forced convection (260 rpm). In the absence of stirring, the anodic current increases progressively with the scan rate, demonstrating a marked dependence on mass transport to the electrode surface. In contrast, when the experiment is performed with forced convection, the voltammetric response remains practically unchanged across the entire range of scan rate evaluated. This behavior indicates that continuous solution renewal minimizes the diffusional limitations associated with the transport of CBD and the species generated during its oxidation, thus reducing the influence of the sweep rate on the electrochemical response (In Figure S3 of the Supplementary Material, the effect of scan rate on the current recorded by CV is shown under steady-state and agitated conditions, compared at 10 mV/s and 500 mV/s). Taken together, these results are consistent with the classical theory of hydrodynamic voltammetry described by Bard and Faulkner [38] and demonstrate that, although surface processes associated with coupling species occur during CBD oxidation, mass transport continues to play a predominant role in the electrochemical response of the system.

**Figure 5:**
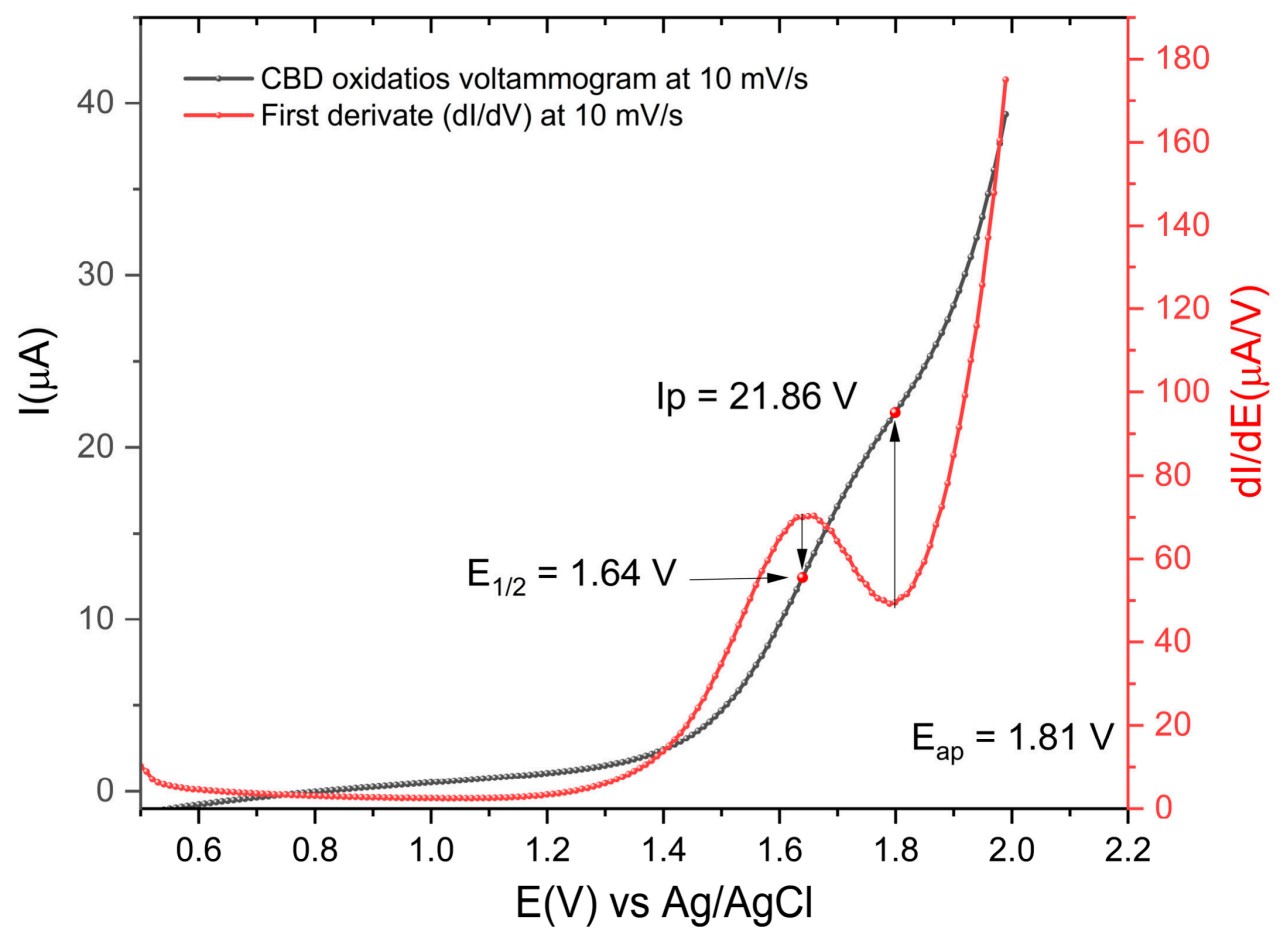
Cyclic voltammogram of CBD oxidation (black line) and its first derivative, dI/dE (red line), recorded at 10 mV/s. The red dots indicate the characteristic points selected from the first-derivative profile according to the changes in curve concavity. These points were used to determine the peak current (*I_p_*) and the half-wave potential (*E*_1/2_), while the anodic peak potential (*E_ap_*) was obtained from the voltammogram.

Under steady-state conditions, mass transport was conditioned by the temporal development of the diffusion layer, which favored the progressive depletion of CBD concentration near the electrode surface. Due to the kinetically irreversible nature of CBD oxidation, the recorded voltammograms did not exhibit a clearly defined current maximum but rather a broad anodic waveform characteristic of irreversible processes with relatively slow electron-transfer kinetics. Under these conditions, direct determination of the peak current (*I_p_*) is imprecise. For this reason, it was determined by calculating the first derivative of the current with respect to potential ($\frac{dI}{dE}$) (Figure 5), a procedure that allows for the objective location of the point corresponding to the maximum current change associated with the oxidation process. The maximum of the first derivative coincides with the experimental half-wave peak potential (*E*_1/2_). After the maximum of the first derivative, where the concavity change occurs, extrapolating to the original curve allows for the experimental determination of *I_p_* and the peak potential (*E_ap_*) in this type of irreversible system. This approach has been previously used to analyze electrochemical processes with similar voltammetric responses, where the absence of a well-defined current maximum makes conventional determination of electrochemical parameters difficult [39,40].

Additionally, it was observed that the peak potential (*E_ap_*) shifted systematically toward more positive values as the scan rate increased (Figure S4, Supplementary Material). This behavior is characteristic of irreversible electrochemical processes, in which the electron transfer rate is not high enough to maintain equilibrium at the electrode–solution interface. Consequently, the system requires a higher overpotential to maintain analyte oxidation at increasing scan rates, resulting in the experimentally observed anodic shift in Ep.

**Figure 6:**
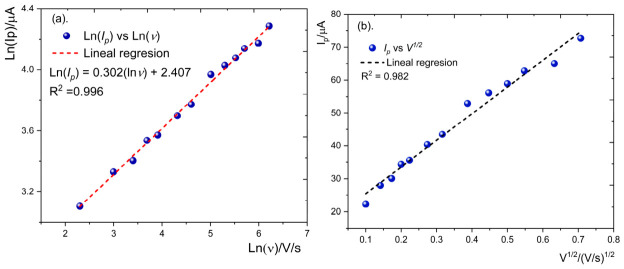
(**a**) Relationship between ln(*I_p_*) and ln(*v*) for the electrochemical oxidation of 150 µM CBD at an FTO electrode in 0.1 M LiClO_4_/ACN. (**b**) Relationship between the anodic peak current (*I_p_*) and the square root of the scan rate (*ν*^1/2^) for the electrochemical oxidation of 150 µM CBD at an FTO electrode in 0.1 M LiClO_4_/ACN.

Analysis of the relationship between peak current and scan rate provides additional information on the mechanism governing the electrochemical oxidation of CBD at the FTO electrode. The plot of ln(*I_p_*) versus ln(ν) (Figure 6a) showed a slope of 0.302, lower than the theoretical value of 0.5 expected for a process controlled exclusively by diffusion. This result indicates that a purely diffusional model cannot describe the electrochemical response; rather, surface phenomena contribute to the overall behavior of the system. However, the linear dependence observed between *I_p_* and ν^1/2^ (Figure 6b) confirms that mass transport continues to play a predominant role during the irreversible oxidation of CBD. Taken together, these results indicate that the electrochemical response is primarily governed by the diffusional transport of CBD molecules to the electrode surface, although modulated by surface processes associated with the products generated during oxidation [41].

This interpretation is consistent with the previously proposed oxidation mechanism for CBD, in which electron transfer leads to the formation of resorcinol group radicals capable of undergoing coupling reactions to generate dimers initially and subsequently oligomeric species [33,42]. These results indicate that these species transiently modify the electrode–solution interface, as evidenced by the progressive decrease in current during the first voltammetric cycles and the recovery of the electrochemical response after cleaning by sonication. These findings suggest that the interaction between the oxidation products and the FTO surface does not lead to the formation of a permanent passivating film (fouling), but rather to a limited and reversible surface modification under the experimental conditions used.

The response observed under forced convection reinforces this interpretation. Continuous homogenization of the solution decreases the local accumulation of species generated during oxidation. It promotes their transport into the bulk of the solution, reducing their residence time at the electrode–solution interface. Consequently, the voltammetric response becomes practically independent of the scan rate, allowing a stable electroactive surface to be maintained during the electrochemical process.

Overall, the experimental evidence obtained suggests that the irreversible oxidation of CBD on FTO follows a mechanism consistent with that previously described for compounds with a resorcinol structure. However, the surface nature of FTO limits the permanent accumulation of coupling products, preventing the development of irreversible passivation like that reported for other electrode materials that require subsequent mechanical cleaning steps [32,43].

Under these conditions, the electrochemical response results from the interaction between a predominant mass transport process and a transient surface modification associated with CBD oxidation products. This explains the electrochemical stability of FTO and supports its use as a platform for developing electrochemical sensors for cannabidiol determination.

In contrast to CV, the voltammetric profiles obtained by DPV showed only a slight decrease in the oxidation current during the first few cycles, followed by a virtually stable response between cycles 8 and 10 and a slight increase thereafter. Overall, the variations remained within a relatively narrow current range, without showing the progressive decrease observed with CV (Figure S5, Supplementary Material). This behavior suggests that, under the measurement conditions used in DPV, the surface modification of the electrode is less pronounced, possibly due to inherent differences between the two voltammetric techniques, such as the shorter effective polarization time and the absence of a continuous potential sweep—factors that could limit the accumulation of oxidation products on the FTO surface.

Once the optimal electrochemical conditions were established, DPV was selected for constructing calibration curves and quantifying CBD in the commercial oil samples. This choice was supported not only by the electrochemical behavior observed during the characterization stage but also by the analytical advantages of the technique [44]. While in CV the recorded current corresponds to the sum of the faradaic current, dependent on the analyte concentration, and the capacitive current associated with the charge of the electrical double layer, in DPV the differential current measurement significantly reduces the capacitive contribution. As a result, a better signal-to-noise ratio, better resolution of the analytical signal, and greater sensitivity are obtained, allowing for more precise quantification of CBD [38].

### 3.1. Electrochemical Determination of CBD

**Figure 7:**
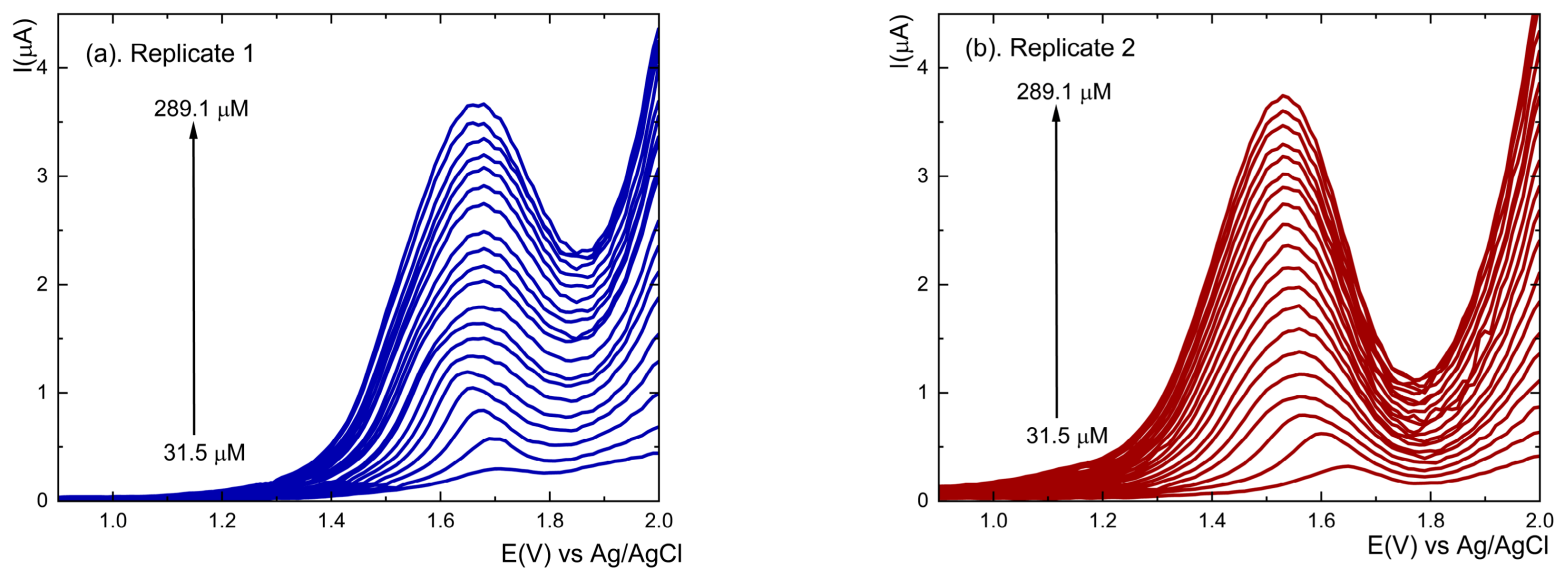

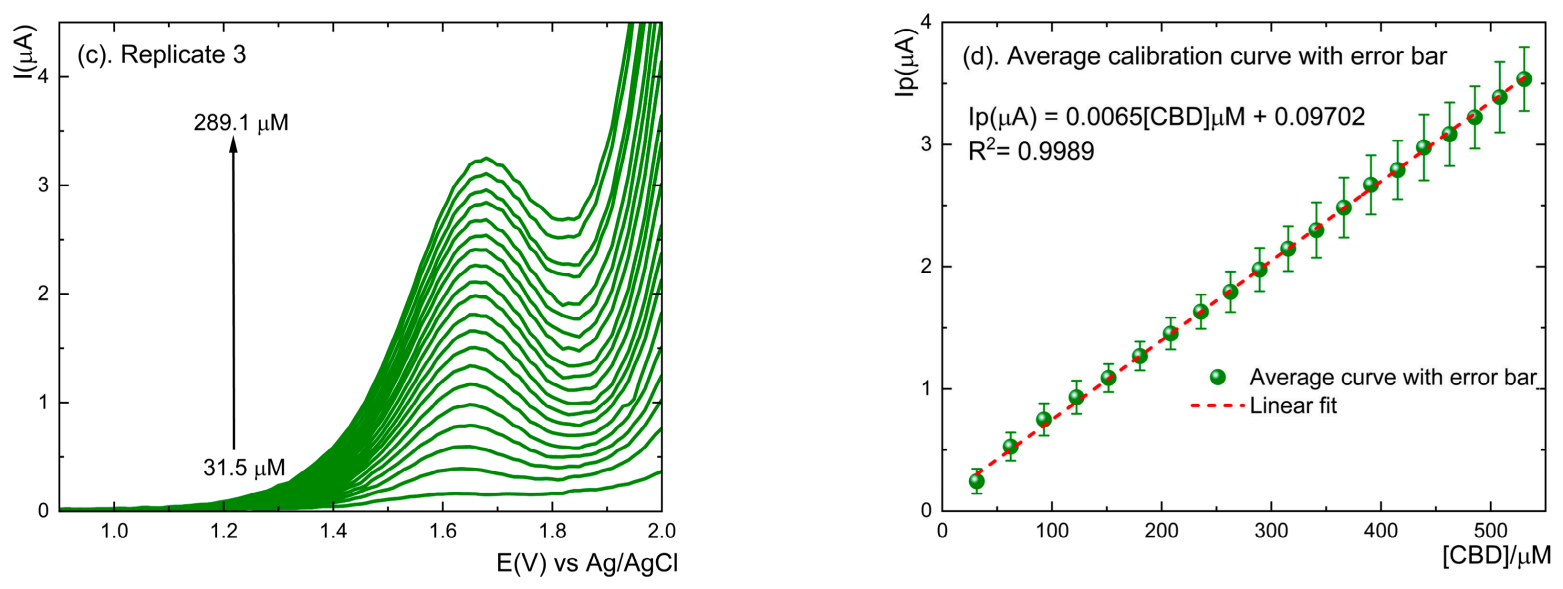
(**a**–**c**) Differential pulse voltammograms recorded during the measurement of a CBD standard, interface: FTO/LiClO_4_ 0.1 mol/L, *n* = 3. (**d**) Average calibration curve with error bar (standard deviation).

In constructing the calibration curve using differential pulse voltammetry on an FTO electrode, a linear response was obtained in the range of 31.5–289.1 µM of CBD (see Figure 7a–c, with replicates carried out for the construction of the calibration curve). The proportionality between the peak current (*I_p_*, µA) and the CBD concentration is evident, with the calibration equation *I_p_* (µA) = 0.0065[CBD]µM + 0.09702 (R^2^ = 0.9989) shown in Figure 7d (calibration curve averaging the I_p_ and its standard deviation at each concentration level). The 20 successive measurements on the same electrode demonstrated high reproducibility, attributable to the chemical and geometric stability of the FTO, which minimizes surface variability during prolonged measurements (Figure S6, Supplementary Material).

### 3.2. Determination of CBD in Commercial Samples

**Figure 8:**
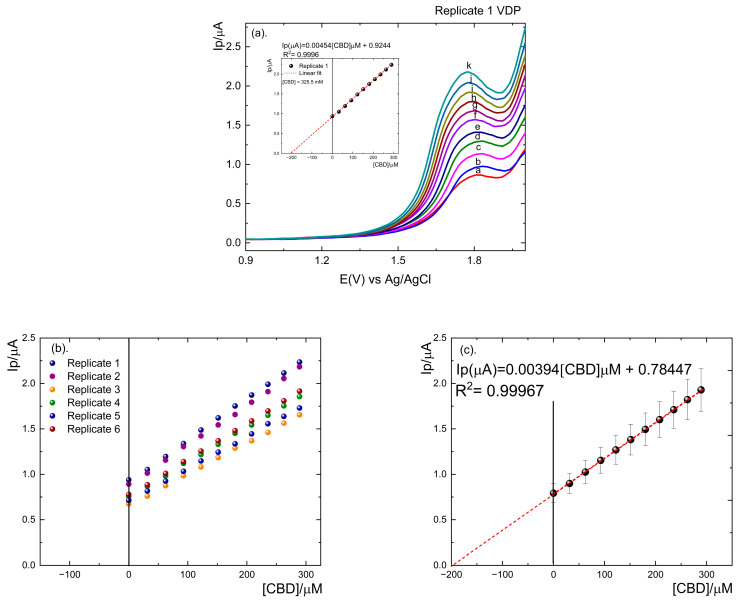
(**a**) Differential pulse voltammograms obtained during the standard addition of CBD in a commercial medicinal cannabis sample (FTO/0.1 mol L^−1^ LiClO_4_). Curves (a–k) correspond to successive additions of CBD, giving final concentrations of 0.0, 31.5, 62.3, 92.5, 122.4, 151.4, 180.0, 208.0, 235.6, 262.7 and 289.1 μM, respectively. The inset shows the linear regression of peak current versus added CBD concentration; the red dashed line corresponds to the regression extrapolated to the x-axis, and the x-intercept was used to calculate the initial CBD concentration in the electrochemical cell. (**b**) Calibration curves for each replicate (*n* = 6). (**c**) Average calibration curve with error bars.

Figure 8a shows the voltammograms obtained using Differential Pulse Voltammetry (DPV) during the application of the standard addition method to the extract of the commercial magistral sample. The red curve corresponds to CBD extracted from the magistral formulation in the electrolyte solution (LiClO_4_ 0.1 M in ACN), which shows a signal associated with the electrochemical oxidation of CBD, with a peak current (*I_p_*) of 0.8690 µA, confirming the presence of endogenous levels of the analyte coming from the lipid sample that was previously dissolved in the solvent system described earlier. Afterward, 10 successive additions of the CBD standard solution (3.2 mM (1.0 mg/mL) in methanol) were made, resulting in a proportional increase in the peak current directly proportional to the amount of analyte added. This linearity in the electrochemical response allowed us to build the specific calibration curve (*I_p_* vs. [CBD] µM) for the sample matrix, thus compensating for potential interferent effects coming from the lipid matrix (see the inset graph in Figure 8a) and obtaining a quantification of the actual CBD content in the commercial formulation. The repetitions carried out (*n* = 6) with the same electrode confirm the stability of the material and the reproducibility of the data obtained (Figure S7, Supplementary Material).

To assess experimental reproducibility and linearity, the results of the 6 replicates analyzed for CBD quantification in the commercial formulation sample were compiled; see Figure 8b. For each additional concentration level of CBD, the average analytical signal and its standard deviation were calculated from the six (6) independent determinations. These values were used to construct the average calibration curve (Figure 8c) and its respective error bars (±standard deviation), as well as the regression line obtained, *I_p_* (μA) = 0.00394[CBD]µM + 0.78447 with a determination coefficient of R^2^ = 0.99967, parameters used only to evaluate the linearity of the analytical response and the stability of the FTO electrode in the concentration intervals and potential disturbances in each of the measurements, and not to estimate the method’s precision. The low spread observed among the curves and the high coefficient of determination obtained from the average curve indicate the consistency of the measurements (stability and reproducibility of the FTO in instrumental responses) within the tested concentration range. These results also demonstrate the robustness of the implemented methodology, ensuring the traceability of measurements and the reliability of CBD concentration measurements in lipid samples.

The same electrode was used, with the same intermediate cleaning protocol described in the methodology, for the six determinations of the analyte content by the standard addition method (*n* = 6) with 11 electrochemical measurements in each repetition, thus ensuring the reproducibility of the measurements and the reliability of both the calibration curve and the analytical determinations performed.

### 3.3. Statistical Comparison Between Electrochemical and HPLC Methodologies

The results and comparative statistical analysis for the determination of CBD by both methodologies are summarized in Table 1. The simultaneous fulfillment of both criteria will allow us to conclude that the electrochemical and reference methodologies are methodologically equivalent, thereby validating the reliability of the results obtained (See the chromatograms obtained for HPLC analysis in Supplementary Material Figure S8).

The results of the comparative analysis obtained from the six independent replicates of each methodology are summarized in Table 2. The comparative statistical analysis between the DPV and HPLC revealed that, for the Fisher test, the calculated F value (F_calculated_ = 1.30) was lower than the tabulated critical value (F_critical_ = 5.05, *α* = 0.05, df_1_ = df_2_ = 5), which indicates that there is no significant difference between the experimental variances of both methodologies; that is, the precision of the electrochemical technique is statistically equivalent to that of the reference method (HPLC). Similarly, the Student’s *t*-test for two experimental means yielded a calculated t-value (t_calculated_ = 0.620) lower in absolute value than the tabulated critical value (t_critical_ = ± 2.228, *α* = 0.05, ν = 10), demonstrating that the observed difference between the means of [CBD] of both methodologies is not statistically significant and can be attributed to indeterminate or random errors. Taken together, the simultaneous fulfillment of both statistical criteria (F_calculated_ < F_critical_ and |t_calculated_| < t_critical_, equivalent to *p* > 0.05 in both tests) corroborates that the electrochemical methodology with a bare FTO substrate and the HPLC methodology provide statistically similar concentration values for the analyzed sample (318 mM or 100 mg/mL of CBD in olive oil). Therefore, the reliability and methodological equivalence are validated, confirming the analytical reliability of the DPV method for quantifying CBD in lipid matrices.

### 3.4. Determination of Limits of Detection and Quantification

The limits of detection and quantification were determined by evaluating peak current variation (*I_p_*) across 10 consecutive replicates of the blank electrolyte solution (LiClO_4_ 0.1 mol L^−1^ in ACN). Thus, a LoD of 4.8 µM and a LoQ of 14.4 µM were obtained.

The LoQ of 14.4 µM confirms that the method not only detects but also reliably quantifies CBD from this level, maintaining a linear relationship between peak current and concentration within the working range. The low dispersion of the signals recorded across replicates indicates a stable and reproducible voltammetric response, suggesting consistent interaction between the analyte and the FTO surface under the experimental conditions used. Taken together, these results demonstrate that the FTO electrode, in the optimized electrolytic medium, provides adequate analytical performance for the detection and quantification of CBD at low levels, with good linearity, accuracy, and sensitivity.

The use of modified electrodes has dominated the detection and quantification of cannabinoids when employing electroanalytical techniques to improve sensitivity and portability, for example: Pholsiri et al. integrated VPD into a capillary microfluidic device with a screen-printed electrode modified with multi-walled carbon nanotubes (SPE-MWCNT-Ag), operating in phosphate-buffered solution at pH 7.0 and demonstrating a wide linear range applicable to cannabis flowers with an LoD of 0.03 µM [17]. Similarly, López-Iglesias et al. [43] proposed adsorptive redissolution differential pulse voltammetry (AdSV-DPV) in an aqueous medium using PEDOT-modified Sonogel–Carbon electrodes, thereby enabling effective quantification of CBD with an LoD of 0.23 µM. They also reported the formation of an insulating layer on the electrode after measurement, reducing the electroactive area of the proposed electrode and requiring mechanical renewal of the electrode surface to minimize fouling phenomena [43]. This has also been reported by other authors [45]; in this regard, the improvement in detection and quantification limits is notable, as are the requirements for obtaining and reproducing the electrode surface on a larger scale, as well as the need for cannabinoid separation processes prior to quantification. In some cases, renewal of the electrode surface is required, which could hinder its use for commercial or routine purposes. Against this backdrop, there has recently been growing interest in the search for simpler strategies that employ electrode substrates without modification, as in the case of Lima et al., who analyzed the potential of unmodified carbon-based electrodes (glassy carbon electrodes (GCE) and commercial screen-printed carbon electrodes (SPCE)) for the electrochemical detection of CBD in pharmaceutical dosage forms, emphasizing the simplicity and high analytical throughput enabled by the absence of sample preparation and electrode modification. Unlike methods that require substrate modification, the use of unmodified electrodes allows for robust and efficient analysis [46]. Table 3 summarizes some recently reported studies using unmodified electrodes.

Given the above, current scientific literature shows a surge in the search for analytical solutions that simplify sample handling while ensuring high reproducibility and practicality in routine laboratory work. The proposed methodology employs completely bare FTO electrodes without any surface modification process, which contributes to this research approach. Methodological simplicity represents a significant advantage for interlaboratory reproducibility, as it eliminates experimental variables associated with the homogeneity of nanostructured coatings, the stability of modifications, and surface renewal protocols. The direct use of intrinsic FTO properties—conductivity, stability in organic media, and surface uniformity—ensures consistent results across different batches of commercial electrodes, maintaining analytical sensitivity in complex medicinal cannabis matrices without compromising methodological robustness. This supports scaling up the methodology for routine analysis and contributes to the quality control of commercial products containing CBD.

The FTO substrate demonstrated a reproducible and quantifiable electrochemical response to CBD. However, this study did not evaluate the sensor’s response to other phytocannabinoids structurally related to CBD, such as THC, CBG, CBN, etc., and/or other compounds with similar structures. Therefore, the possibility of cross-sensitivity to these analytes in the proposed electrochemical system cannot be ruled out. Consequently, the results reported here should be interpreted as an FTO response to CBD in CBD-OS formulations prepared with CBD isolate, where other phytocannabinoids are not expected to be present.

## 4. Conclusions

In this work, an analytical methodology for the quantification of CBD in an oral pharmaceutical formulation (CBD-OS) was presented, employing an unmodified FTO electrode and electroanalytical techniques. The redox behavior of CBD on the FTO surface was evaluated through a detailed study using cyclic voltammetry, which demonstrated irreversible oxidation of the analyte governed by the diffusion of CBD molecules towards the electrode surface without irreversible modification of the FTO in the presence of forced convection.

The proposed analytical methodology involved the use of DPV as a quantification technique, which allowed for stable and reproducible measurements, with a LoD of 4.8 µM and a LoQ of 14.4 µM. Linear ranges were determined between 31.5 and 289.1 µM, with a high correlation between the instrumental signal and the analyte concentration (R^2^ = 0.9989). The proposed methodology was used to quantify CBD in a pharmaceutical sample (CBD-OS). Using standard addition to the diluted oil sample, six independent measurements were performed, and the results were validated against conventional HPLC methodology. Statistical analysis corroborates that the electrochemical methodology with an unmodified FTO substrate and the HPLC methodology provide statistically similar concentration values for the analyzed sample (318 mM of CBD in olive oil or 100 mg/mL).

The results obtained demonstrate the feasibility of using an unmodified FTO electrode with intrinsic properties to create a simple, reproducible, robust, and cost-effective analytical methodology for the analysis of CBD in pharmaceutical samples.

## Figures and Tables

**Table 1:** Results and comparative statistical analysis for the determination of CBD using the electrochemical DPV and the reference HPLC methodologies.

Parameters	[CBD] mM (DPV)	[CBD] mM (HPLC)
Replicate 1	325.5	320.9
Replicate 2	317.4	323.0
Replicate 3	316.3	319.4
Replicate 4	311.7	311.5
Replicate 5	319.2	321.6
Replicate 6	314.8	318.0
Experimental media ($\bar{X}$)	317.5	319.1
Standard deviation (s)	4.7	4.1
Variance (s^2^)	21.8	16.8
[CBD] ± $t\cdot \frac{s}{\sqrt{n}}$ $t_{p=0.05}^{df=5}=2.015$	317.5 ± 3.9	319.1 ± 3.4

**Table 2:** Results of statistical tests applied to determine the CBD content in commercial pharmaceutical samples.

Test	Statistic	Calculated Value	Tabulated Critical Value	Decision
Test (F)	$F=\frac{s_{1}^{2}}{s_{2}^{2}}$	1.30	F (0.05, df_1_ = 5 df_2_) = 5.05	Equivalent variances
Test (t)	$\left|t\right|=\frac{\left({\bar{X}}_{1}-{\bar{X}}_{2}\right)}{s_{p}\cdot \sqrt{\left(\frac{1}{n_{1}}-\frac{1}{n_{2}}\right)}}$ $s_{p}^{2}=\frac{\left(n_{1}-1\right)\cdot s_{1}^{2}+(n_{2}-1)\cdot s_{2}^{2}}{\left(n_{1}+n_{2}-2\right)}$	0.620	t (0.05, 10 df) = 2.228	Statistically equal experimental means

*n* = 6 replicates per methodology; df = degrees of freedom; s = standard deviation.

**Table 3:** Comparative analytical properties of published electrochemical methods for CBD using unmodified electrodes.

Analyte	LoD/µM	LoQ/µM	Electrode	Medium	Reference
CBD	0.38	1.11	Unmodified GCE	B-R 0.1 mol L^−1^, pH 7.0:EtOH (75:25 *v*/*v*)	[46]
CBD	2.72	-	Unmodified SPE	NaOH 0.5 mol/L	[47]
CBD	15.92	48.24	Unmodified SPE	PBS pH 12	[48]
CBD	4.8	14.4	Unmodified FTO	LiClO_4_/ACN (0.1 M)	This Study

B-R: Britton–Robinson buffer, SPE: Carbon Screen-Printed Electrode, GCE: Glassy Carbon Electrode.

## Data Availability

The data that supports this research can be found in the Supplementary Material.

## Supplementary Materials

The following supporting information can be downloaded at: https://www.mdpi.com/article/10.3390/s26154893/s1, Figure S1: Differential pulse Voltammograms with CBD at 0.3 mM using different working electrodes, LiClO_4_ 0.1 mol/L in ACN; Figure S2: Differential pulse voltammograms of CBD 0.3 mM using FTO as the working electrode: (a) evaluating LiClO_4_ 0.1 mol/L in different solvents, ACN and ethanol; (b) in different electrolytes, LiClO_4_ and TBAPF_6_ (0.1 M) in ACN; Figure S3: Cyclic voltammograms recorded during the irreversible oxidation of CBD at scan rates of 10 and 500 mV/s under steady-state conditions and with forced convection at 260 rpm. 150 µM CBD in 0.1 M LiClO_4_/ACN; Figure S4: First derivative of the anodic sweep segment recorded in cyclic voltammetry during CBD oxidation at scan rates of 10, 20, 30, 40, 50, 75, 100, 150, 200, 250, 300, 400, and 500 mV/s. Peak potential shift as scan rate increases; Figure S5: (a) Electrochemical oxidation of 150 µM CBD at the CBD/FTO interface by cyclic voltammetry (25 replicates) in 0.1 M LiClO_4_/ACN by DPV, (b) DPV responses of the FTO electrode before CBD oxidation (black), after CBD oxidation (red), the 25th cyclic voltammogram recorded during the electrochemical oxidation of CBD (green), and after ultrasonic cleaning (magenta). The inset shows an enlarged view comparing the voltammetric responses of the FTO electrode before CBD oxidation and after ultrasonic cleaning; Figure S6: Replication of the calibration curves obtained by DPV, *n* = 3; Figure S7: Recorded differential pulse voltammogram (DPV) of replicates made during standard addition of CBD to a commercial medicinal cannabis sample (*n* = 6), interface: FTO/LiClO_4_ 0.1 mol/L, and calibration curve (Insert). (a) 0.0000; (b) 0.0099 (31.4 µM); (c) 0.0196 (62.3 µM); (d) 0.0291 (92.5 µM); (e) 0.0385 (122.4 µM); (f) 0.0476 (151.4 µM); (g) 0.0566 (180.0 µM); (h) 0.0654 (208.0 µM); (i) 0.0741 (235.6 µM); (j) 0.0826 (262.7 µM) and (k) 0.0909 (289.1 µM); mg/mL of CBD; Figure S8: Chromatograms recorded in the chromatographic measurements to determine the CBD content in the pharmaceutical sample, *n* = 6.

## Abbreviations

The following abbreviations are used in this manuscript:

CBD	Cannabidiol
LoD	Limit of Detection
LoQ	Limit of Quantification
FTO	Fluorine-doped tin oxide
HPLC	High-performance liquid chromatography
df	Degrees of freedom
DPV	Differential Pulse Voltammetry
I_p_	Peak current
