# Supplementary material for: Differential Pulse Voltammetric Analysis of Cannabidiol on Fluorine-Doped Tin Oxide (FTO) Electrode Interface: Quantification in Pharmaceutical Oil with Assessment of Electrode Surface Stability
Source: Sensors (Basel). 2026 Aug 3;26(15):4893. doi: 10.3390/s26154893 (PMC13469201; doi:10.3390/s26154893)
Supplement: Supplementary file 1 [file sensors-26-04893-s001.zip › sensors-4430162-supplementary.pdf]

Differential Pulse Voltammetric Analysis of Cannabidiol on Fluorine-Doped Tin Oxide (FTO) Electrode Interface: Quantification in Pharmaceutical Oil with Assessment of Electrode Surface Stability

Mario Romero [1] , Eliana Rocha Fontalvo [1], Maria L. Ospina-Castro [1] , Victoria A. Arana [2] , Carlos Mario Melendez [3], Jolian Andres Vargas Alzate [4] and Andrea Ramos-Hernandez [1],*

[1] Grupo de Investigacion Quimica Supramolecular Aplicada, Programa de Quimica, Facultad de Ciencias Basicas, Universidad del Atlantico, Carrera 30 No. 8-49, Puerto Colombia 081007, Colombia; marioromero@mail.uniatlantico.edu.co (M.R.); emariarocha@mail.uniatlantico.edu.co (E.R.F.); mariaospina@mail.uniatlantico.edu.co (M.L.O.-C.)
[2] Grupo de Investigacion Ciencias, Educacion y Tecnologia—CETIC, Programa de Quimica, Facultad de Ciencias Basicas, Universidad del Atlantico, Carrera 30 No. 8-49, Puerto Colombia 081007, Colombia; victoriaarana@mail.uniatlantico.edu.co
[3] Grupo de Investigacion en Quimica Organica y Biomedicina, Programa de Quimica, Facultad de Ciencias Basicas, Laboratorio de Investigacion en Biologia Molecular, Universidad del Atlantico, Carrera 30 No. 8-49, Puerto Colombia 081007, Colombia; carlosmelendez@mail.uniatlantico.edu.co
[4] CUBIKAN GROUP SAS, Finca Veracruz, Vereda La Diamantina—km 10 via La Argelia, Ansermanuevo 762018, Colombia; info@cubikangroup.com

* Correspondence: andrearamos@mail.uniatlantico.edu.co

Supplementary materials

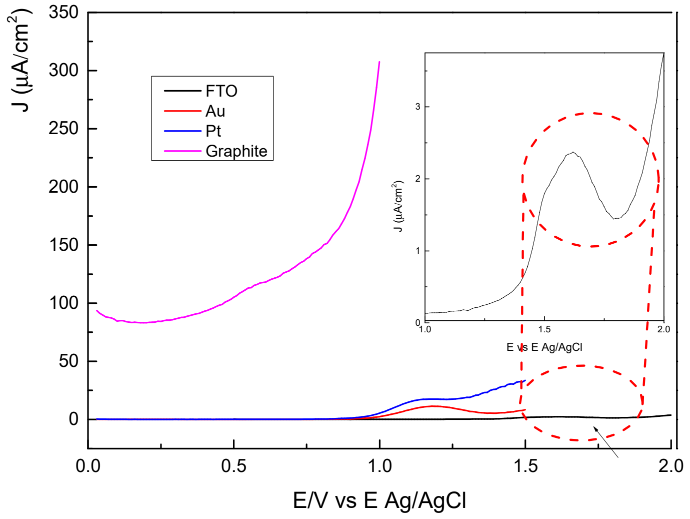

Figure S1. Differential pulse Voltammograms with CBD at 0.3 mM using different working electrodes, $LiClO_4$ 0.1 mol/L in ACN.

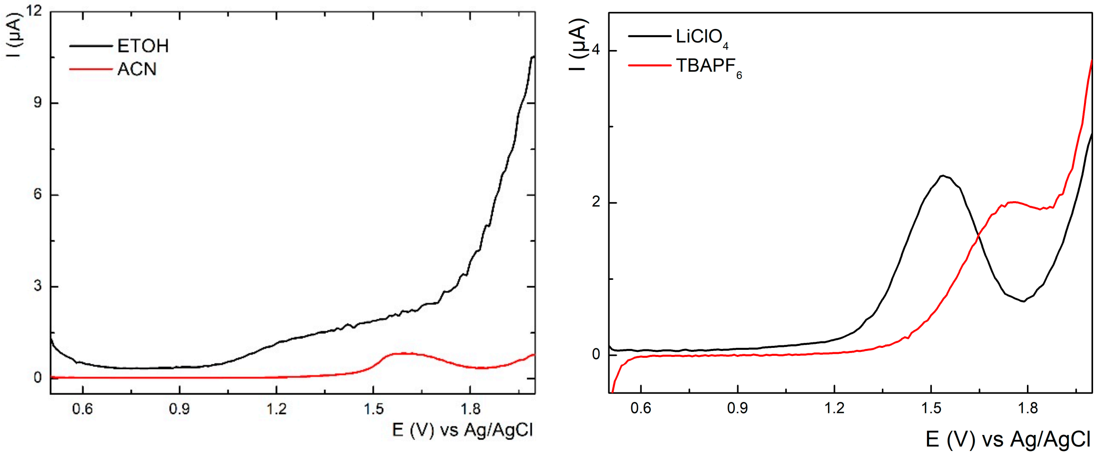

Figure S2. Differential pulse voltammograms of CBD 0.3 mM using FTO as the working electrode: a) evaluating $LiClO_4$ 0.1 mol/L in different solvents, ACN and ethanol; b) in different electrolytes, $LiClO_4$ and $TBAPF_6$ (0.1 M) in ACN.

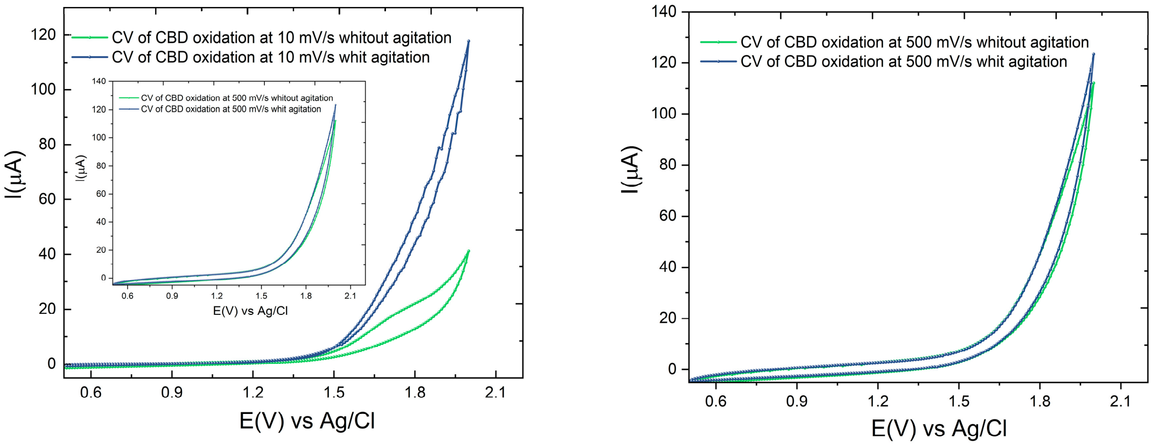

Figure S3: Cyclic voltammograms recorded during the irreversible oxidation of CBD at scan rates of 10 and 500 mV/s under steady-state conditions and with forced convection at 260 rpm. 150 μM CBD in 0.1 M LiClO4/ACN

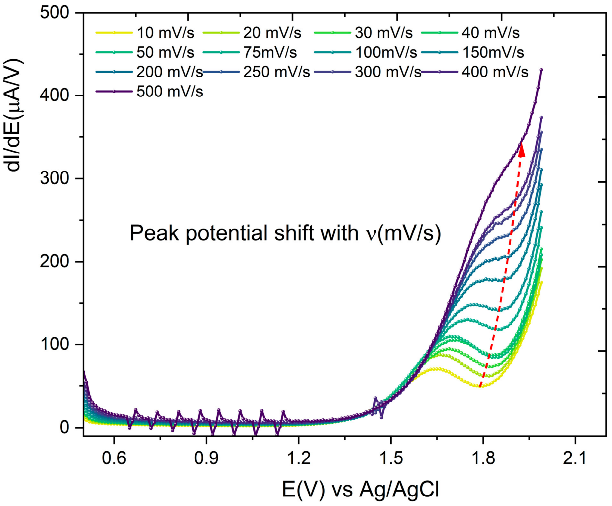

Figure S4. First derivative of the anodic sweep segment recorded in cyclic voltammetry during CBD oxidation at scan rates of 10, 20, 30, 40, 50, 75, 100, 150, 200, 250, 300, 400, and 500 mV/s. Peak potential shift as scan rate increases

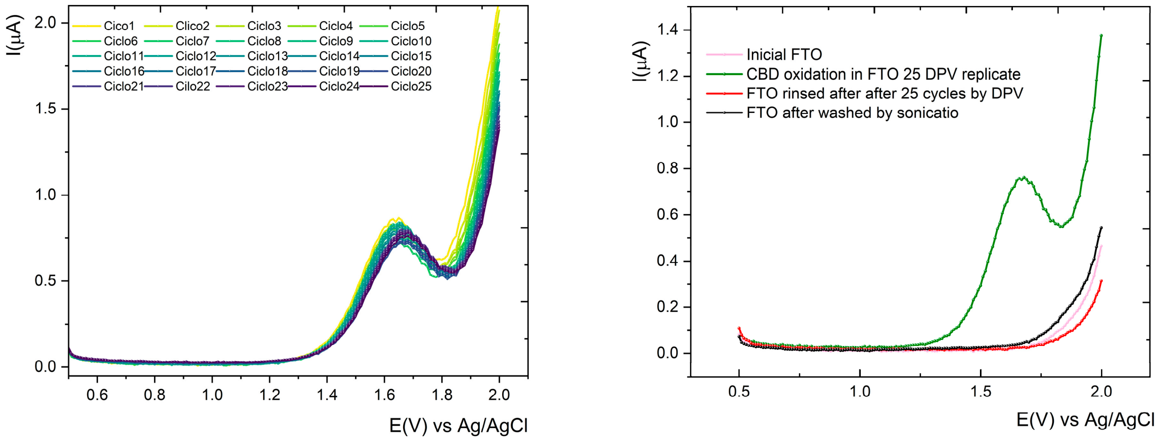

Figure S5. (a)Electrochemical oxidation of 150 μM CBD at the CBD/FTO interface by cyclic voltammetry (25 replicates) in 0.1 M LiClO4/ACN by DPV, (b) DPV responses of the FTO electrode before CBD oxidation (black), after CBD oxidation (red), the 25th cyclic voltammogram recorded during the electrochemical oxidation of CBD

(green), and after ultrasonic cleaning (magenta). The inset shows an enlarged view comparing the voltammetric responses of the FTO electrode before CBD oxida-tion and after ultrasonic cleaning

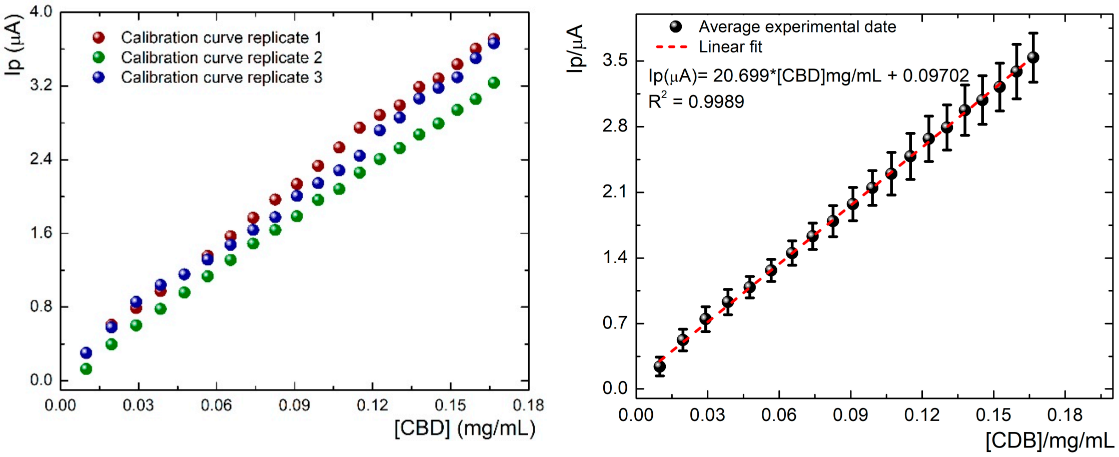

Figure S6. Replication of the calibration curves obtained by DPV, *n*=3.

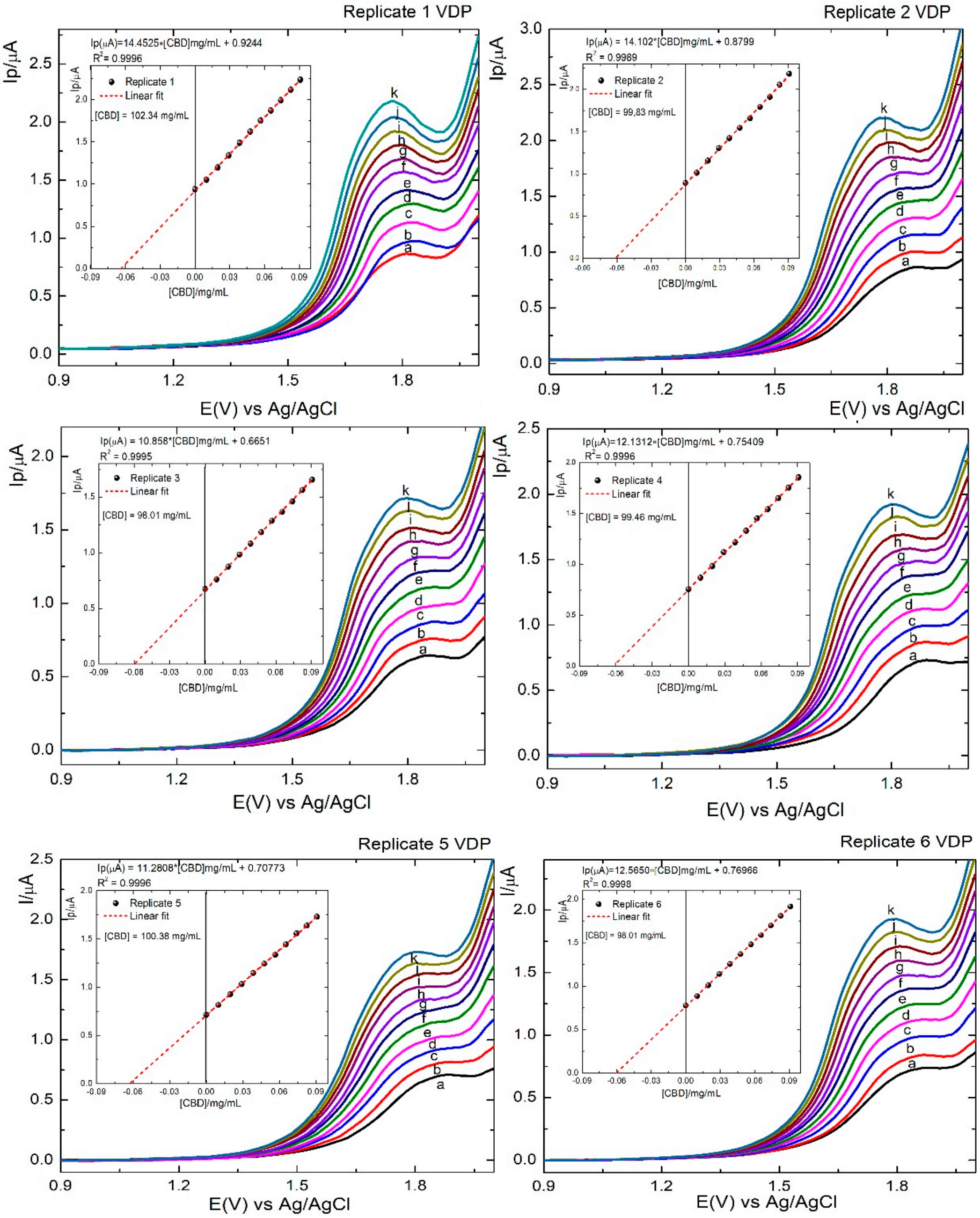

Figure S7. Recorded differential pulse voltammogram (DPV) of replicates made during standard addition of CBD to a commercial medicinal cannabis sample (n = 6), interface: FTO/LiClO4 0.1 mol/L, and calibration curve (Insert). (a) 0.0000; (b) 0.0099 (31.4 μM); (c) 0.0196 (62.3 μM); (d) 0.0291 (92.5 μM); (e) 0.0385 (122.4 μM); (f) 0.0476 (151.4 μM); (g) 0.0566 (180.0 μM); (h) 0.0654 (208.0 μM); (i) 0.0741 (235.6 μM); (j) 0.0826 (262.7 μM) and (k) 0.0909 (289.1 μM); mg/mL of CBD.

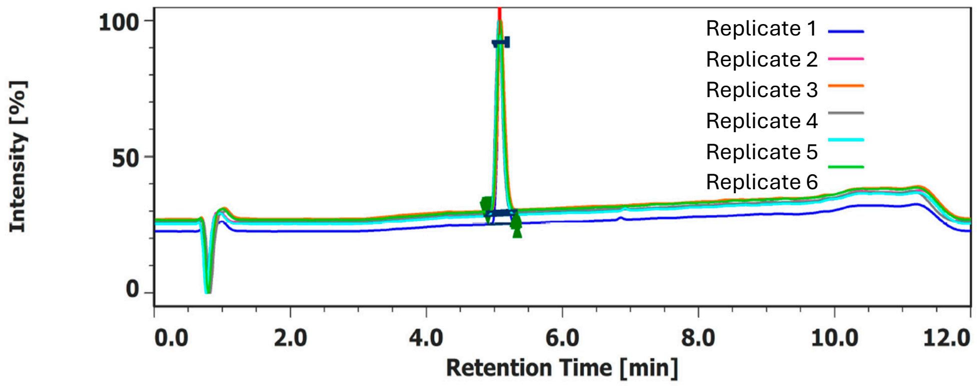

Figure S8. Chromatograms recorded in the chromatographic measurements to determine the CBD content in the pharmaceutical sample, *n*=6.
